# Focusing with colorectal cancer patients: a pilot study of a brief online group intervention

**DOI:** 10.3389/fpsyg.2024.1339823

**Published:** 2024-08-08

**Authors:** Marta Gomes, Eunice R. Silva, João Salgado

**Affiliations:** ^1^Department of Social and Behavioural Sciences, University of Maia, Maia, Portugal; ^2^Center for Psychology, University of Porto, Porto, Portugal; ^3^Portuguese Institute of Oncology of Porto Francisco Gentil, EPE, Porto, Portugal

**Keywords:** focusing, clearing a space, cancer patients, mental health distress, psychological wellbeing

## Abstract

**Introduction:**

Focusing-Oriented Psychotherapy has had a long history and influence on the field of psychotherapy. By “clearing a space” and “focusing,” individuals can enhance their emotional awareness and improve their ability to self-regulate. These tasks are particularly relevant in the context of Psycho-Oncology, although the research on their potential benefits for cancer patients is limited. Furthermore, the application of these tasks in a group or online setting has not been thoroughly explored.

**Methods:**

This study aimed to examine the effectiveness of a two-session online intervention based on Focusing for cancer-diagnosed participants and its impact on their mental health and wellbeing. The study involved three participants with a diagnosis of colorectal cancer who were undergoing palliative treatment. We used both qualitative and quantitative methods. PFC-2 was used to assess participants' accomplishment of the task; FMS was used to assess the change in the focusing attitude, while CORE-OM, and PWBS-RV were used as mental health distress and psychological wellbeing measures; participant feedback was collected through questionnaires and a semi-structured interview.

**Results:**

The results suggest that the tasks led to greater self-awareness, heightened self-reflection, and a sense of relief for the participants.

**Discussion:**

These findings suggest that the group intervention protocol based on online Focusing sessions is potentially useful for broader applications.

## 1 Introduction

Cancer poses a significant global concern due to its high prevalence and mortality rates. The diagnosis of cancer can be a potentially traumatic experience for individuals, given its impact on their functionality and the life-threatening nature of the disease (Cordova et al., 2017). Throughout the course of the illness, individuals often face numerous challenges, including intense emotional reactions, heightened vulnerability, concerns about the disease, treatments, and mortality, as well as disrupted sleep, appetite, and a sense of losing control (Holland et al., 2010).

Consequently, living with cancer often leads to intense emotional distress, and mental disorders (Mitchell et al., 2011; Mehnert et al., 2018). The intensity of these distress states can be significantly influenced by the coping strategies individuals choose to employ. Individuals tend to resort to avoidance and emotional suppression as coping mechanisms, despite the evidence indicating their ineffectiveness and inappropriate nature in effectively managing emotionally painful experiences (Bauer et al., 2017; Stanton et al., 2018).

According to models of emotional processing, an adaptive approach to dealing with emotionally painful experiences involves a gradual process comprising several stages: activation, awareness, symbolization, and reflection of emotions (Greenberg, 2002; Greenberg and Pascual-Leone, 2006). These subprocesses facilitate the transformation of the painful experience into an assimilated one that encompasses both affective and cognitive aspects. Any interruption or interference with this process can lead to difficulties, increasing the likelihood of employing dysfunctional strategies. This is coherent with the studies on cancer that reveal that such difficulties have been associated with psychological distress and psychopathology (Stanton et al., 2018; Guimond et al., 2019; Baziliansky and Cohen, 2021) and adverse effects in physiological health (Schlatter and Cameron, 2010). Conversely, the ability to express and regulate emotions seems to have a positive impact on adapting to cancer (Brandão et al., 2016).

In order to provide effective support for people coping with cancer, it may be important to consider approaches that could promote emotional awareness and regulation. However, the diversity of empirically supported interventions is still limited, and these approaches are still not strongly linked to specific interventions. This study aims to evaluate the feasibility of a promising intervention at this level: focusing. This task seems particularly useful in the context of Psycho-Oncology, although studies on its potential benefits for supporting cancer patients remain scarce. The purpose of the present study is to examine the experience gained from a two-session online intervention based on Focusing with cancer-diagnosed participants, as well as its association with mental health and wellbeing variables.

### 1.1 Focusing

Focusing-Oriented Psychotherapy has a long history and influence in the field of psychotherapy. In general, Focusing can be used to clarify and improve emotional contact, aimed at improving self-regulation and emotional awareness (Gendlin, 1996). On one hand, Focusing has developed as a specific form of psychotherapy; on the other hand, given its broad applicability, it also influenced other neo-humanistic and experiential models, which directly or indirectly incorporated some of its insights and techniques in their theory and practice.

Emotion-Focused Therapy is one of the approaches that integrates Focusing and Clearing a Space (CAS) as specific therapeutic tasks (Elliott et al., 2004; Greenberg, 2014). Focusing is, more than a technique, a specific attitude that focusing-oriented therapists aim to foster with their clients, constituting a main block for the therapeutic change (Cornell, 1996; Gendlin, 1996; Rappaport, 2009). In EFT, it has been used as therapeutic task among others, and it involves naming and clarifying the bodily sensations experienced, to achieve a sense of relief and/or new meanings (Elliott et al., 2004; Greenberg, 2014, 2021). The aim is for the client to describe their sensations using words, images, or expressions that symbolize and create meaning for their experience, leading to a sense of relief and increased awareness of what is being experienced (Elliott et al., 2004; Greenberg and Watson, 2006; Greenberg, 2014, 2021).

By its turn, CAS was originally introduced as one of the preparatory steps to initiate Focusing, as it was considered useful in helping the client to achieve a state of focus and regulate their emotions in the present moment (Gendlin, 1996). However, over time, CAS has been explored as an independent task, and there have been a few studies investigating its independent application with different populations (Klagsbrun, 2007; Klagsbrun et al., 2010). Nonetheless, its use in combination with the Focusing task remains relevant, and CAS can be used as a preparation for effective Focusing work.

### 1.2 Focusing and the adaptation to cancer

Focusing appears to be particularly valuable in the Psycho-Oncology context. On one hand, the CAS intervention focuses on developing the ability to establish a “working distance” from emotionally distressing objects, specifically any thoughts, concerns, memories, or experiences that evoke strong negative emotions or cause psychological discomfort/distress. On the other hand, Focusing then aims to sustain focused attention on these objects, facilitating activation, awareness, symbolization, and reflection on the underlying emotions (Gendlin, 1996; Elliott et al., 2004; Greenberg and Watson, 2006; Greenberg, 2014, 2021). These two processes are promising paths to promote better adjustment to such a potentially traumatic event.

There are direct and indirect evidence supporting this claim. First, there are three studies supporting Focusing and/or CAS utility when applied to cancer patients (Katonah and Flaxman, 1991; Klagsbrun et al., 2005, 2010). In Katonah and Flaxman's (1991) study, 12 cancer patients, aged between 31 and 55, participated in a six-week training using Focusing, specifically the CAS technique. The results showed that Focusing led to a significant reduction in depression and improved body attitudes among the participants. Moreover, the participants reported experiencing reduced fear of dying and positive behavioral changes in self-care. These improvements were sustained over a six-month follow-up period. This work also suggests that the Focusing may be a promising psychosocial intervention to aid in the recovery and adjustment of cancer patients, providing valuable support for their emotional wellbeing and overall health (Katonah and Flaxman, 1991). In the study by Klagsbrun et al. (2010), the CAS task promoted a greater sense of calm, better emotional self-regulation, coping strategies, increased mental clarity, general wellbeing, and a sense of empowerment in the way of dealing with fear, anxiety, and cancer-related issues. Furthermore, CAS has demonstrated applicability at distance, via a telephone intervention (Klagsbrun et al., 2010). This task, when associated with expressive arts, also led to an increase in the quality of life and improvement in body image in the study conducted by Klagsbrun et al. (2005), which included 18 female participants diagnosed with breast cancer.

There are also some indirect evidence of the potential benefit of these two technics coming from research exploring Focusing as a facilitator of the work process for experiences related to traumatic situations (Leijssen, 2007; Santen, 2014), post-traumatic stress disorders (Coffeng, 2005), dissociative disorders (Coffeng, 2005; Krycka, 2010), and cases of trauma with destructive behavioral patterns (Gunst and Vanhooren, 2018). These studies have shown that Focusing promotes the connection with bodily sensations, allowing the client to safely observe and ultimately alter how these experiences are perceived within their own body (Leijssen, 2007). Other researchers have also studied the use of these techniques in stress management contexts (Klagsbrun, 2007; Rinaldi et al., 2019). In a pilot study conducted by Rinaldi et al. (2019), focusing showed a significant reduction in stress among healthcare professionals and an enhanced receptiveness to internal experiences. Furthermore, Klagsbrun (2007) identified the CAS task as a brief yet effective tool for stress reduction.

However, studies on the application of CAS and Focusing to cancer patients are still very scarce and preliminary, limited in number and with small samples, so more robust research is needed. This becomes even more relevant, since in recent years, studies on interventions targeted at the oncology population have increased (Teo et al., 2019; Carlson, 2023); however, the diversity of interventions supported by empirical evidence is still limited, which restricts the range of choices available to patients (Carlson, 2023). Due to the high influx of people in need of psychological support and the limited resources of healthcare systems, it is not always possible to provide a prompt response to patients (World Health Organization, 2022). As a result, there is a growing need to promptly address the mental health difficulties faced by individuals. Creating and implementing patient-centered, brief psychological interventions could serve as a valuable solution to address these needs. The creation of more forms of intervention at the emotional regulation level becomes important to increase the range of potential choices available to patients, since different people have different preferences when it comes to emotional regulation techniques (e.g., Vanderlind et al., 2020). Focusing has demonstrated positive indicators, and it can be useful for people who want to clarify and improve contact with their emotions for better self-regulation and emotional awareness. Once learned, CAS and Focusing can be a helpful resource both inside and outside of the therapeutic context.

The implementation of brief, online, and group interventions can potentially be useful for the oncology population and for healthcare systems themselves, as they involve low economic costs, require few human resources, and do not require specific physical spaces. Advances in technology and the pandemic situation had created the opportunity and the need to use digital tools for mental health. Digital interventions have been studied regarding their effectiveness, potentialities, and limitations (Andersson, 2018; Kemp et al., 2020; Willems et al., 2020), and the practice of Focusing on an online format could be a potential resource for mental health professionals and institutions. Additionally, the use of groups in the oncological context is particularly important for promoting emotional expression, sharing experiences, and social support (Watson and Kissane, 2011). This allows individuals who are going through a cancer diagnosis to feel a sense of belonging and explore new ways to deal with their adaptation to the disease (Ussher et al., 2006; Kissane and Ngan, 2015).

The present study aims to investigate the effect of two focusing sessions applied in a group context, and in an online format on the level of emotional clarity and psychological wellbeing of cancer patients undergoing palliative treatment for colorectal cancer. According to the latest data provided by The Global Cancer Observatory (2021), colorectal cancer is the third most common cancer diagnosis globally and the most prevalent type in Portugal among men and women. Patients diagnosed with colorectal cancer often experience intense physical symptoms (Holtedahl et al., 2021), undergo aggressive treatments and face significant side effects (Vonk-Klaassen et al., 2016), resulting in elevated levels of distress (Abelson et al., 2018). Over the course of the disease, more than 50% of patients develop metastases, and palliative treatment is often used to reduce symptoms and prolong life (Greer et al., 2013; Kurk et al., 2018).

We aim to investigate the experiences of participants in an online intervention consisting of two focusing practice sessions, as well as the relationship between focusing practice and indicators of mental health and wellbeing. Because we are dealing with patients with potentially high levels of emotional vulnerability, we believe that new methods of intervention need to start with very careful and very small sample testing in a way that ensures patients safety – as is done in the area of safety studies for biomedical interventions (Wright, 2017). Thus, this pilot study will involve a small number of patients to allow for careful evaluation of the interventions' potential safety and usefulness, personalized monitoring of each patient's progress, and preparation for larger study in the future. The intervention protocol will be assessed based on feasibility indicators such as adherence to intervention, the appropriateness of the intervention for these patients (as determined by focusing indicators, wellbeing indexes, clinical symptoms of mental health, and feedback from participants); the suitability of study instruments, and analysis from both quantitative and qualitative perspectives.

## 2 Materials and methods

### 2.1 Participants

Participants were recruited from a cancer hospital. Inclusion criteria included being diagnosed with metastatic colorectal cancer, receiving palliative treatment at the hospital, with no occurrence of disease progression or changes to the therapeutic plan. Eligibility criteria further included being over 18 years old, having adequate computer literacy to fill out online forms and having internet access. Exclusion criteria included the presence of severe psychopathology (e.g., major depression; anxiety disorders with an elevated level of dysfunctionality associated) or dementia, as well as changes in the psychopharmacological treatment plan in the last month. The total sample consisted of 3 female participants aged between 30 and 59 years. All participants were employed.

### 2.2 Procedures

**Figure 1:**
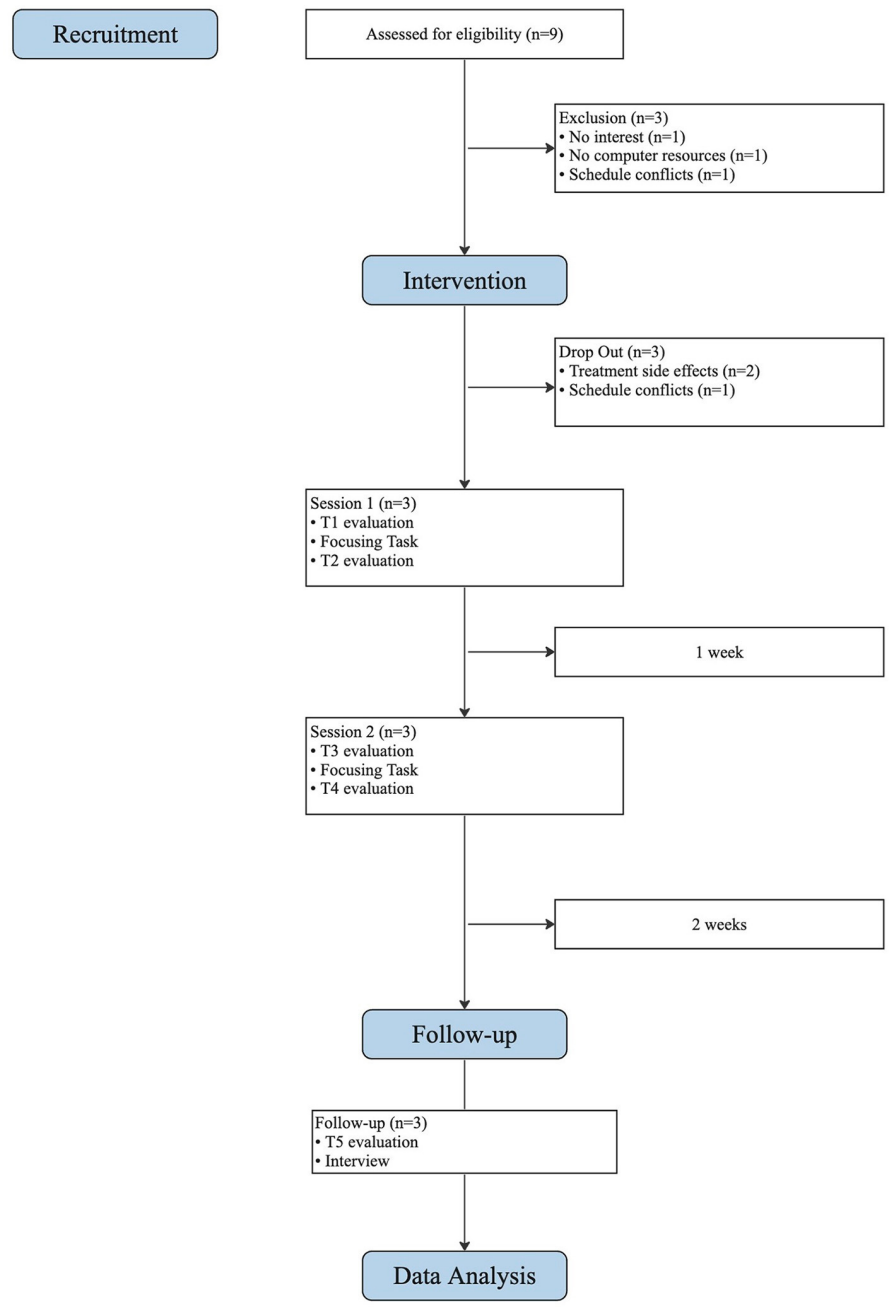
CONSORT flowchart of intervention process.

Ethical approval from the hospital's institutional committee was obtained before the study's initiation. Participants were recruited from the hospital's psychology consultation with the assistance of two hospital psychologists. All individuals who met the criteria for the study were considered potential participants and were contacted with an equal probability of being included in the study. Of the nine potential participants contacted, six expressed interest and met the inclusion criteria. However, three of the six participants dropped out before the first session, resulting in a final sample of three participants. All participants were provided with a detailed description of the intervention and its ethical considerations and signed informed consent forms. Participants completed a screening evaluation and were subsequently assessed at various points during the intervention. Two weeks after the second session, a follow-up evaluation was conducted, which included the completion of a questionnaire and a semi-structured interview with each participant. The interviews lasted between 10 and 15 min and focused on the participant's overall experiences. Figure 1 illustrates the flowchart of the process in a schematic and concise form.

### 2.3 Intervention

The group intervention protocol was based on two online focusing sessions. Each session lasted 90 min and was separated by 1 week. The tasks included the “clearing space” and “focusing” stages outlined by Elliott et al. (2004) to help participants find a safe distance to access and regulate their emotions. Participants completed the tasks in a self-guided manner and were provided with an audio guide to complete the task at home. Participants had the opportunity to share their experiences, provide feedback and reflect on the potential utility of these tasks for the future. It is important to note that one of the participants required additional support from the therapists due to the emotional activation experienced after the practice of the tasks.

Table 1 provides a comprehensive overview of the intervention sessions, while Table 2 offers a concise description of the stages involved in the tasks utilized.

**Table 1:** Intervention sessions.

**Session**	**Week**	**Duration**	**Description**
1	1	90 min	Introductions of participants
			Group procedures (e.g., confidentiality; privacy)
			Intervention aims
			Structure of the sessions
			Guided practice of clearing a space and focusing task
			Reflection and sharing of experiences on task performance
			Homework: perform the task at least once more at home using audio instructions
2	2	90 min	Group procedures highlight
			Homework review
			Guided practice of clearing a space and focusing task
			Conclusion of the intervention: reflection on the session and recommendations for the future

**Table 2:** Task stages.

**Stage**	**Description**	**Task instructions**
Task initiation	Identifying worries: instructions to help participants in identifying worries and compiling a list of three.	“During the task, we're going to start by asking you to identify worries that make you uncomfortable and difficult to deal with. These are the ones we'll work on. I'm going to ask you to make a list of three worries.”
	Grounding technique: encourage participants to adopt a comfortable seated position and do breathing exercises to prepare for the upcoming task.	“Please, be seated in a comfortable position. You can close your eyes or keep them open, as you prefer. If you choose to keep your eyes open, we invite you to focus on a point around you.” “Take three deep breaths. Inhale through the nose and exhale through the mouth.”
Clearing a space	Listing worries and creating distance: guide participants through the process of identifying worries and imaginatively setting each one aside to a designated place.	“What is preventing me at this moment from feeling completely well?” “Pay attention to what worries you or makes you feel discomfortable.” “Now imagine putting these worries or feelings at some distance from yourself.” “For example, you can place it in the corner of the room, in a box…any place you find better.”
	Appreciating cleared internal space: encourage participants to take a few moments to explore the sensation when worries are away from their internal space.	“Once you have set the worries aside, pay attention to the middle part of your body, and see ≪how are you feeling right now≫?”
Focusing	Identify the primary concern: prompt participants to select the worry that causes the most discomfort and direct their attention inward.	“Now I'll ask you to choose one worry to focus on. Choose the one that causes you the most discomfort and bring it close. Focus on what you feel in your body remembering each aspect of this worry.”
	Exploring descriptive labels: encourage participants to search for descriptive words, labels or mental images that capture the sensation experienced in their body and verify if it fits.	“Now, I would like you to try to find a word, a quality that best describes the sensation in your body.” “Check in your body if this image, phrase, or name fits”
	Appreciating the resulting sensation: guide participants to briefly hold the sensation.	“How are you feeling right now? Hold that sensation for a few seconds.”

### 2.4 Therapists

The sessions were conducted by the first author, a master's in clinical and health psychology and supervised by a certified emotion-focused therapist, who is also a clinical and health psychologist in the hospital (second author). Before and throughout the intervention process, the first author received specific training sessions as well as supervision.

### 2.5 Measures

#### 2.5.1 Sociodemographic data collection

Sociodemographic data sheet. This questionnaire was used to evaluate generic sociodemographic data such as gender, age range and main activity.

#### 2.5.2 Focusing practice

Focusing Manner Scale (FMS; Aoki and Ikemi, 2014). This is a 25-item questionnaire which measures the focusing attitudes. The present study translated to European Portuguese language the English version of FMS. Participants were asked to answer with a Likert scale scored on 4 points (from 1 = *never*; to 4 = *frequently*). Participant total scores could range from 25 to 100. The English version of FMS had α coefficient of.75 (Aoki and Ikemi, 2014).

Post-Focusing Questionnaire-2 (PFC-2; Alemany, 1986). This is a 13-item self-assessment questionnaire with a dichotomic response option (*Yes/No*) that aims to evaluate the participant's ability to focusing. The lower the score, the better the focusing ability. This measure demonstrated a good overall internal consistency (α = 0.789) (Alemany, 1986), and we used a version translated to European Portuguese language.

#### 2.5.3 Mental health and wellbeing

Clinical Outcome Routine Evaluation—Outcome Measure (CORE-OM; Evans et al., 2000; Portuguese Version by Sales et al., 2012). This is a 34-item self-report instrument that measures mental health distress of adults. Items intend to evaluate four main domains: (1) subjective wellbeing; (2) social and personal functioning; (3) problems and symptoms; and (4) risky behavior (for self and/or others). Items are rated on a five-point Likert scale that ranges from 0 (*Never*) to 4 (*Always, or almost always*). Scores can range from 0 to 40, and values between 0 and 10 correspond to subclinical mental health distress, 10–14 correspond to mild mental health distress, 15–19 correspond to moderate mental health distress, 20–24 correspond to moderate to severe mental health distress and values between 25 and 40 correspond to severe mental health distress (Barkham et al., 2013). The Portuguese version of CORE-OM had α coefficient of 0.94 (Sales et al., 2012).

Clinical Outcome Routine Evaluation−10 (CORE-10; Barkham et al., 2013; Portuguese Version by Sales et al., 2012). This is a reduced version of CORE-OM, composed of 10 items. The values are interpreted on a scale of 0–4, and only the sum of the items are performed. The results can be interpreted similarly to those of CORE-OM. This version was used only at the screening moment, while the full version (CORE-OM) was used in the remaining moments of evaluation.

Psychological Wellbeing Scale—Reduced Version (PWBS-RV; Ryff, 1989; Portuguese version by Ferreira and Simões, 1999). This is an 18-item subjective wellbeing assessment instrument that assesses parameters related to self-acceptance, positive relationships with others, autonomy, environmental dominance, meaning of life and personal growth. The answers are assigned using a Likert scale ranging from 1 (*I completely disagree*) to 6 (*I completely agree*). The Portuguese version of this measure had α coefficient of 0.94 (Ferreira and Simões, 1999).

#### 2.5.4 Qualitative measures

End-of-session questionnaire. These questionnaires were applied at the end of each session to record the participant's feedback. There were open questions about the session's experience, the task implementation, and suggestions for improvement. The questionnaires further included an assessment of satisfaction and usefulness of each session through a Likert scale ranging from 1 (*Not satisfied/useful)* to 5 (*Very satisfied/helpful)*.

Semi-structured interview. A semi-structured interview was conducted with each participant at the end of the intervention. The aim of the interview was to obtain feedback regarding the entire intervention process through open questions (e.g., *How was your experience in the participation of this intervention; What was your experience on the focusing practice*).

The measures were applied at different evaluation moments (see Table 3) in an online form created in Limesurvey, a software designed for the purpose of applying questionnaires, which ensures the security and proper treatment of data. After inclusion in the study, each participant was assigned an alphanumeric code to safeguard their personal data.

**Table 3:** Measures and evaluation moments.

**Measures**	**Evaluation moments**					
	**T0**	**T1**	**T2**	**T3**	**T4**	**T5**
Sociodemographic questionnaire	X					
CORE-OM		X		X		X
CORE-10	X					
PWBS-RV		X				X
FMS		X			X	X
PFC-2			X		X	
End-of-session questionnaire			X		X	
Final process interview						X

T0 = Screening; T1 = Evaluation before first session; T2 = Evaluation after first session; T3 = Evaluation before second session; T4 = Evaluation after second session; T5 = Evaluation after 15 days of T4 (follow up).

### 2.6 Data analysis

Data analysis was performed in a mixed way, i.e., quantitative analyses (e.g., calculations of magnitude post-test effect) and qualitative analyses, based on thematic analysis methods (Braun and Clarke, 2006) of the final process interview and feedback's questionnaires at the end of each session. Qualitative analyses allow a more personalized characterization of the personal experience and the change processes that may be set in motion. The collected data was analyzed using the Excel version 16.0 program of Office 365 for Microsoft Windows and Jamovi version 2.2.5 for Microsoft Office.

## 3 Results

### 3.1 Results of participant adherence

Initially, the intervention included 6 participants who completed the first evaluation at screening moment. However, prior to the first session, three participants dropped out due to reasons such as cancer diagnosis and its related side effects and emotional aspects, and schedule conflicts.

### 3.2 Pre-assessment scores

The results prior to the first session are presented in Table 4. According to the results of the CORE-OM, “Ana” presented mild mental health distress, while participants “Sara” and “Olivia” revealed moderate and moderate to severe mental health distress, respectively. Overall, the T1 results indicated mental health difficulties ranging between mild and severe levels, as expected. “Olivia” was signaled as potentially more vulnerable than the other two, and specific attention to her results was carried out.

**Table 4:** Scores obtained before first session (T1).

**Participants**	**CORE-OM**	**PWBS-RV**	**FMS**
“Ana”	10	103	78
“Sara”	16	83	67
“Olivia”	20	69	71

The participants general psychological wellbeing scores were obtained using the PWBS-RV instrument. “Ana” had the highest score in terms of psychological wellbeing and “Olivia” the lowest score in comparison to the other participants. The FMS measure was used to evaluate focusing attitudes present in each participant. “Ana” showed the highest score, while participant “Sara” revealed the lowest score in this measure.

### 3.3 Outcome assessment: focusing measures

The PFC-2 and FMS are the main outcome measures of the study as they evaluate the measures related to focusing. The PFC-2 was applied at the end of each session to evaluate the focusing capacity of each participant (see Table 5). The scores obtained at the end of the first session (T2) demonstrated that all participants could perform the focusing process. At the end of the second session (T4), “Ana” and “Sara” reported again a remarkably elevated level of focusing. “Olivia” reported slightly lower level when compared with the other two participants, and with her own level of focusing on the previous session; nevertheless, her self-reported results indicate a partial ability to perform the focusing process on that session.

**Table 5:** PFC-2 scores.

**Participants**	**Moment**	
	**T2**	**T4**
“Ana”	2	2
“Sara”	2	1
“Olivia”	5	7

**Figure 2:**
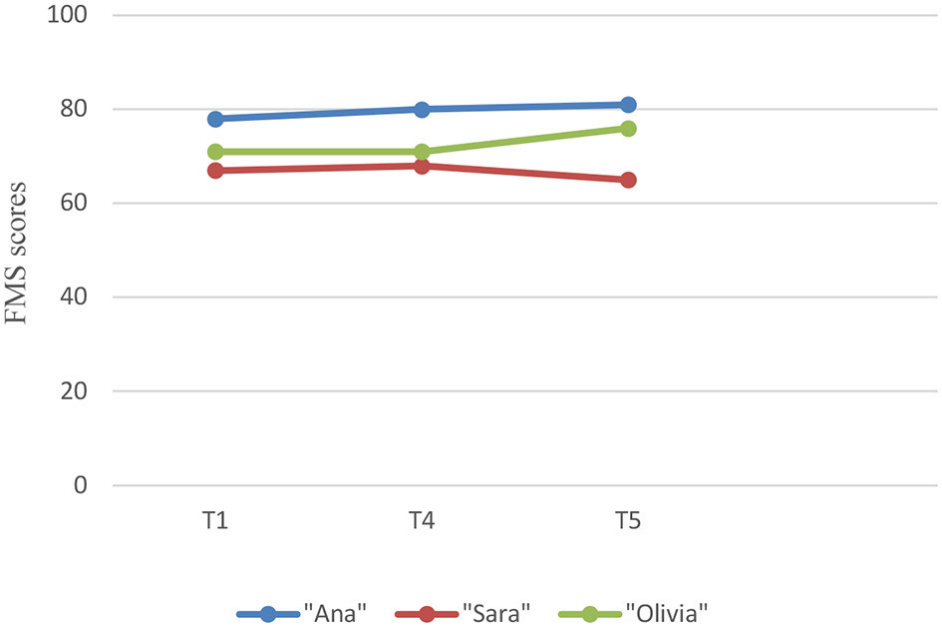
Evolution of focusing attitudes per participant (FMS).

The FMS was applied to evaluate the presence of focusing attitudes during the therapeutic process. The focusing attitudes displayed subtle variations: “Ana” and “Olivia” had an increase in their level of focusing attitudes and “Sara” had a decrease in her level of focusing attitudes (see Figure 2). This change occurred in the desired direction, presenting a moderate to high effect size (Cohen's d = −0.555).

### 3.4 Outcome assessment: mental health and wellbeing measures

**Figure 3:**
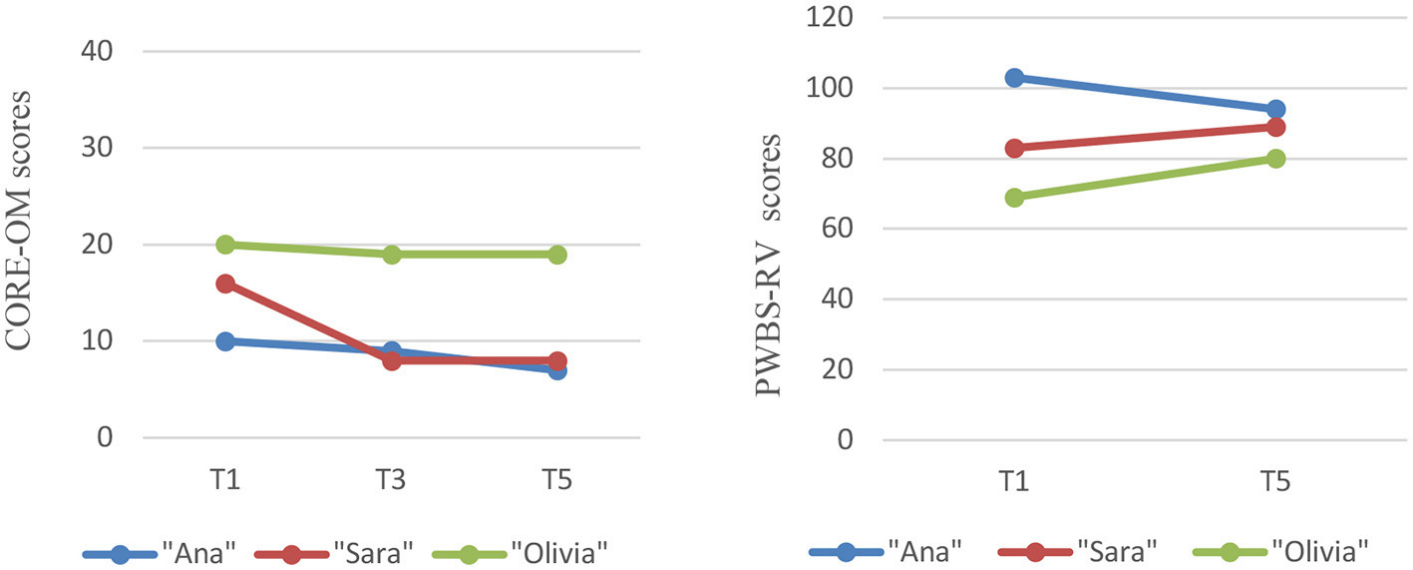
Mental health and wellbeing changes per participant.

Throughout the intervention process, an analysis of mental health assessment and psychological wellbeing measures was conducted, in addition to the focusing measures. All participants showed a slight decrease in CORE-OM scores. “Sara” and “Olivia” increased their psychological wellbeing sightly over the intervention period, while “Ana's” level declined. Figure 3 presents the mental health and wellbeing changes per participant.

Effect size, measured through Cohen's d, was calculated for each of these measures. The change observed in the CORE-OM was in the desired direction, presenting a high effect size (CORE-OM Cohen's d = 1,272). The PWBS-RV presented the lowest effect size compared to the other instruments; however, the change was still in the desired direction, showing a small to moderate effect size (Cohen's d = −0.256).

### 3.5 Outcome assessment: qualitative measures

The qualitative analysis of the intervention revealed three domains that represent the participant's perspective: Autonomous Use of the Task; Results Achieved; and Intervention Process. The data were gathered from the final process interview and answers to open questions in the end-of-session questionnaires. Table 6 presents the themes for each domain.

**Table 6:** Summary of domains and themes.

**Domain**	**Theme**		***N***
Autonomous use of the task	Autonomy as positive		2
	Difficulties with the task		2
Results achieved	Positive results	Self-knowledge Awareness/reflection Feelings of relief	2
	Negative results	Negative emotions Overwhelming feelings	1
Intervention process	Satisfaction		2
	Positive evaluation of the protocol		2
	Improvement suggestions		1

#### 3.5.1 Autonomous use of the task

Autonomous Use of the Task emerged as a domain. Through the analysis of the participants' discourse, it was possible to note that the autonomous task performance enhanced the personalization of tasks. We identified Autonomy as Positive as a theme highlighted by a participant, “Sara”, who specifically valued the possibility of using the tasks in an autonomous and even personalized way (“*... memorized more or less and do without the audio...”*; “*... I do the task mentally... in a shorter and faster way, but what is certain is that... gives me tranquility”*), including the creation of personalized meaning while distancing from concerns in the CAS practice (“*... put them in the green bag of hope...”*; “*... it's not just putting them in some corner, but taking them out and delivering them...”*). Two participants also mentioned the advantage of being able to perform the task at their own pace [“*... the advantage is... I stop... stop at that problem..*.(“Olivia”)”; “*... as you do it, it seems that you clarify and, and accept better* (“Olivia”)”; “*... I can even be, for example, in a break, in a space, waiting for an appointment and then I close my eyes and so fast... step through this essential core* (“Sara”)”]. On the other hand, two participants reported difficulties with focus throughout the task, specifically focusing on the body and distancing from the concerns in CAS task [“*Lack of concentration and focus. Many problems to arise at the same time* (“Olivia”)”; “*... this part of the focus on the body... I don't think I did it completely well..*.(“Sara”)”].

#### 3.5.2 Results achieved

This second domain (Results Achieved) was subcategorized into positive results and negative results. Two participants, “Ana” and “Sara” identified only positive results, while “Olivia” identified mixed results. Positive Results were identified through gains in self-knowledge [“*... it was to get to know myself a little more...”*; “*... general form of self-knowledge...”* (“Ana”)], awareness, reflection [“*... allowed me to become aware..*. (“Sara”)”; “*to put concerns into perspective and list them...”; “... was to relive all experiences before and after diagnosis* (“Olivia”)”; “*... more confidence in myself, in what I truly feel, without the fears of expressing them..*.(“Ana”)”], and feelings of relief [“*... way that makes us feeling lighter, with peace... that helps us to rebalance* (“Sara”)”; “*... in the end a good, light, sweet feeling..*. (“Sara”)”; “*I cried a lot and after crying... I'm more relieved..*.(“Olivia”)”]. However, negative results were expressed by “Olivia” through negative emotions (“*... very painful for me”*; “*... shuffled and sad.”*; “*... heavy...”*), and overwhelming feelings (“*All these feelings together, it seemed like i was inside a tornado and couldn't get out of there.”*).

#### 3.5.3 Intervention process

The Intervention Process revealed that participants had a general Satisfaction with the intervention [“*... was positive in every way..*. (“Ana”)”; “*... very good, rewarding, useful experience..*.(“Sara”)”; “*Enriching..*. (“Sara”)”]. The Positive Evaluation of the Protocol was also highlighted, especially regarding the online format of the intervention [“*... we created empathy there, even through a screen..*.(“Ana”)”; “*... for this type of work facilitates... it is a great advantage, we can saved on travel, it is in our context... in our space... very positive..*. (“Sara”)”], the group format [“*... know other stories..*.(“Ana”)”; “*... sharing of emotions..*.(“Ana”)”; “*... sometimes we could be a bit confused or blocked there on an issue and maybe with the sharing of other colleagues we were unlocking it* (“Ana”)”], and the completion of different questionnaires [“*... I didn't think it was too many..*.(“Olivia”)”; “*... made me try to understand myself better really..*.(“Ana”)”]. Some Improvement Suggestions were made by one participant, “Sara”, related to the audio provided to guide the task (“*I would only increase the sound of the voice and start and/or end with music.”*), and related to complementing communication (“*Complementing communication with a WhatsApp group.”*).

## 4 Discussion

This study must be seen as a small, modest step in gaining a better understanding of the potential role of focusing as a tool to help cancer patients cope with their illness. A first reason for this has to do with the exceptionally small sample size that we have used. It is quite impossible and unethical to draw general conclusions on the basis of such a small sample of three patients. However, when introducing innovative treatments, we need to proceed very carefully, using more individual studies before moving to larger trials. This study was designed to explore the feasibility and acceptability of the intervention among cancer patients. As such, our findings provide insights into participants early experiences and lay the groundwork for future research.

The primary aim of this study was to explore the feasibility of a two-session online intervention for participants with a cancer diagnosis, analyzing the participant's experience and the relationship of the practice of focusing with other variables of mental health and wellbeing. By focusing on the feasibility of the intervention, we aimed to assess its potential suitability for wider application and identify areas for improvement to optimize its effectiveness in supporting cancer patients' mental health and wellbeing.

The results of one of the specific focusing measures—PCF-2—suggest that two of the participants were clearly able to do the focusing practice on the sessions (“Ana” and “Sara”), while one participant (“Olivia”) only partially performed the task. The practice of the tasks allowed the participants to achieve positive results such as self-knowledge, awareness, self-reflection, feelings of relief and peace—which are consistent with the literature (Katonah and Flaxman, 1991; Klagsbrun et al., 2010). However, during the intervention process, we identified another type of experience and more complex results than those mentioned above. During the first session, “Olivia” had trouble in distancing herself from her concerns, which resulted in emotional distress and an inability to detach from the pain caused by her concerns. Due to the high emotional burden involved in the tasks, this participant was asked to stay with the therapists at the end of the first session and then, with this supplemental help, she was able to perform the CAS task individually, to regulate her emotions and meet her immediate needs. Thus, after she performed the task accompanied individually by the therapists, she expressed feelings of relief at the end. These findings suggest that this participant—and probably many others—may benefit more from personalized and individual support; or at least, that in group interventions this possibility of additional individual support right after the group meeting may need to be anticipated as part of the protocol. One explanation for what happened with this participant was her clinical mental health distress, whose score was higher than those of the other participants. It is always important to remember that for this specific population there may be a high number of concerns associated with distress (Holland et al., 2010; Mehnert et al., 2018) which can easily influence the emotional burden involved in performing the tasks. Still, even reporting a painful and complicated process, the participant was able to feel relief at the end, when individually guided by the therapists. This study highlights the importance of a suitable therapist-participant ratio and working in small groups to enable personalized and individualized support during the intervention.

Measures of mental health and wellbeing were analyzed over the course of the intervention. Individual differences in psychological wellbeing emerged, although all participants showed a slight decrease in mental health scores (CORE-OM). Both “Sara” and “Olivia” demonstrated a slight increase in psychological wellbeing. This finding is noteworthy, given “Olivia's initial difficulties in engaging with the focusing practice and her elevated levels of mental health distress. “Ana” experienced a decrease in her psychological wellbeing scores. However, “Ana's” initial psychological wellbeing score was higher than that of the other participants, and despite the decline, “Ana's” final score remains higher than the others. This indicates that although “Ana” experienced a decline, her overall level of psychological wellbeing remains relatively high compared to her peers. It's important to recognize that participants with initially high wellbeing scores may have less room for improvement than those with lower baseline scores. We also found that the effect sizes were in the intended direction.

These findings, together with the individual analysis, may suggest that the intervention is moving in the desired direction by potentially maintaining or even improving participants' mental health and wellbeing. Nevertheless, it is important to interpret the observations with caution, given the limitations of the study, including the small sample size. In addition, although the effect sizes obtained suggest promising indications, it is also necessary to be cautious in their interpretation. Small sample sizes can lead to less precise results, which may affect the accuracy of Cohen's d effect size estimates (Bradley et al., 2002; Leon et al., 2011). Given this variability, caution should be exercised when interpreting effect sizes. It's also important to emphasize that the primary aim of our pilot study was to assess feasibility, not effectiveness. Therefore, while effect sizes provide initial insights, they should not be over-interpreted as conclusive evidence, particularly in pilot studies (Thabane et al., 2010; Leon et al., 2011). Future research with larger samples and robust designs is needed for validation.

The analysis of the intervention protocol as whole suggests that it could be potentially useful for more people. Of the 9 participants contacted, 3 did not enter the study, 3 dropped out before the beginning of the sessions, and 3 remained in the intervention. After the beginning of the sessions, there were no dropouts, revealing a good adherence to the intervention by the participants. However, it is important to consider the reasons for non-integration into the study and previous dropouts, which included personal issues, secondary effects of the treatments, and some participant's vulnerable emotional states or feelings. There were some interested in this intervention but given the periodicity of treatments and the daily hassles of personal life, it was not possible for some people to start the intervention. Additionally, emotional factors related to a cancer diagnosis may make it challenging for people to participate in this type of intervention (Sandaunet, 2008; Savioni et al., 2022). Thus, working with this specific population requires considering potential dropouts or difficulties in forming groups.

The group format provided an important support base for the participants. As participants shared their experiences and offered feedback on the tasks, a strong connection to their cancer diagnosis became evident, highlighting the group's role in providing mutual understanding and support. This is evidence that group support can be a valuable factor for this population, which aligns with the existing literature (Ussher et al., 2006; Watson and Kissane, 2011; Kissane and Ngan, 2015). As for the fact that the intervention was carried out in an online format, this does not seem to hinder the experience of the tasks. Feedback from the participants and the results obtained in the PFC-2 indicated that they were able to perform the tasks and integrate the intervention experience. “Olivia” was only able to perform the task partially, which does not seem to be associated with the online format but rather with their mental health distress. This suggests that it can be possible to apply the focusing practice at distance, which can bring additional advantages in terms of costs and displacements that can be significant for this population (Klagsbrun et al., 2010).

The intervention protocol consisting of two online group sessions appears to be feasible, i.e., potentially useful for wider application. Nonetheless, it would benefit from modifications to better address the specific needs of cancer patients, including extending the number of sessions and implementing a screening process to determine whether a group or individual format best suits the subjective needs of each patient.

Beyond the reduced sample size previously discussed, the present study has also some other limitations that must be considered. Firstly, the study sample consisted entirely of participants of a single gender limits the representativeness of the findings. Additionally, given that this was a pilot study, the results should be interpreted with caution and further replication with a control group is necessary to increase the reliability of the findings. Although the intervention in online format was well-received by participants, it presented some limitations in terms of technical difficulties and computer literacy skills. To address these concerns, future studies could increase the number of sessions to allow more time to cover the crucial parts of the sessions and to optimize the potential benefits of the intervention. Finally, incorporating group dynamics that foster interaction and support among participants could be a valuable addition to future interventions.

## Data availability statement

The original contributions presented in the study are included in the article/supplementary material, further inquiries can be directed to the corresponding author.

## Ethics statement

The studies involving humans were approved by Health Ethics Committee Portuguese Institute of Oncology of Porto FG, EPE. The studies were conducted in accordance with the local legislation and institutional requirements. The participants provided their written informed consent to participate in this study.

## Author contributions

MG: Conceptualization, Formal analysis, Investigation, Writing – original draft, Writing – review & editing. ES: Investigation, Supervision, Writing – review & editing. JS: Methodology, Project administration, Writing – review & editing.
